# Long‐Lasting Hydrogen Evolution and Efficient Dew Harvest Realized via Electrospinning Polyvinylidene Fluoride Membrane on Hybrid Hydrogels

**DOI:** 10.1002/smsc.202400046

**Published:** 2024-05-08

**Authors:** Jie Yu, Mengmeng Chen, Neng Hu, Weijia Wang, Lin Lei, Huiqing Fan, Peter Müller‐Buschbaum, Qi Zhong

**Affiliations:** ^1^ Key Laboratory of Intelligent Textile and Flexible Interconnection of Zhejiang Province & Key Laboratory of Advanced Textile Materials & Manufacturing Technology, Ministry of Education Zhejiang Sci‐Tech University 928 Second Avenue Hangzhou 310018 China; ^2^ State Key Laboratory of Solidification Processing School of Materials Science and Engineering Northwestern Polytechnical University Xi'an 710072 China; ^3^ TUM School of Natural Sciences Department of Physics Chair for Functional Materials Technical University of Munich James‐Franck‐Str. 1 85748 Garching Germany

**Keywords:** dew harvests, electrospinning polyvinylidene fluoride (PVDF) membranes, hybrid hydrogels, Janus structures, photocatalytic hydrogen evolutions

## Abstract

Long‐lasting hydrogen evolution and efficient dew harvest is realized via electrospinning a polyvinylidene fluoride (PVDF) membrane on hybrid hydrogels embedded with photocatalytic g‐C_3_N_4_/Pt nanosheets. Due to the hindrance of water evaporation by the hydrophobic PVDF membrane, the drying process of the hybrid hydrogels significantly slows down. Hence, the g‐C_3_N_4_/Pt nanosheets can continue working on photocatalytic splitting of the water molecules in the hydrogels. When the thickness of the PVDF membrane is 48 μm, the hydrogen evolution rate can reach 2,543 μmol h^−1^ g^−1^, which is 38% more than that of the hybrid hydrogel without covering. Therefore, the hybrid hydrogels covered with PVDF membrane are able to work with high efficiency for 12 h, sufficient for hydrogen evolution during the daytime. In addition, the hydrophobic PVDF membrane and hydrophilic hydrogels construct a Janus structure and induce a fast transport of water molecules from the hydrophobic to hydrophilic side. It is beneficial for the rapid collection of dew in the morning. Based on the long‐lasting hydrogen evolution and efficient dew harvest, the present hybrid hydrogels covered with PVDF membrane are very suitable for the environment rich in solar resource and lack of water supply, such as desert or prairie.

## Graphical abstract

Long‐lasting hydrogen evolution and efficient dew harvest are realized via electrospinning a polyvinylidene fluoride membrane on hybrid hydrogels embedded with photocatalytic g‐C_3_N_4_/Pt nanosheets.

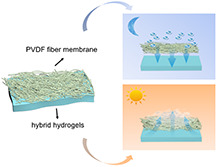

## 1 Introduction

With the development of society, energy shortage has become one of the most important issues we must face at present.^[^ ^1^, ^2^, ^3^, ^4^ ^]^ Hydrogen‐based energy has the advantages of high energy density, strong sustainability, abundant reserves, and zero pollution. Therefore, it is assumed as a green, clean, and sustainable energy source, and plays an important role in solving global energy and environmental problems.^[^ ^5^, ^6^, ^7^, ^8^, ^9^, ^10^, ^11^, ^12^ ^]^ Replacing conventional metal oxide photocatalysts with non‐precious metal materials is an effective way.^[^ ^13^ ^]^ Compared with the other approaches to obtained hydrogen, photocatalytic water splitting simply uses solar energy to obtain hydrogen from water. Thus, it is a green and eco‐friendly way, attracting more and more attention recently.^[^ ^14^, ^15^, ^16^, ^17^, ^18^ ^]^ Unlike the other traditional metal oxide photocatalysts, graphitic carbon nitride (g‐C_3_N_4_) has been broadly investigated in the last decades due to its narrower band gap, which is very suitable for activation via visible‐light illumination.^[^ ^19^, ^20^, ^21^, ^22^, ^23^, ^24^ ^]^ However, as a powder, g‐C_3_N_4_ tends to aggregation and forms large clusters. When dispersed in water, it will quickly sediment from water, which significantly reduces the surface activity sites and lowers the photocatalytic efficiency.^[^ ^25^, ^26^ ^]^

Recently, our group successfully introduced g‐C_3_N_4_ nanosheets into polymer‐based hydrogels.^[^ ^27^, ^28^, ^29^, ^30^, ^31^ ^]^ The additional hydrogel segments not only increase the activity sites via physical separation of the g‐C_3_N_4_ nanosheets, but also enhance the absorption of UV and visible light by the multiple scattering of incident light in the hydrogels. In addition, the large amount of water stored in the hybrid hydrogels can be used as a reservoir for photocatalytic water splitting.^[^ ^25^, ^31^ ^]^ Thus, the obtained hybrid hydrogels can be applied in a nonaqueous atmosphere, such as a desert. However, the hydrophilicity of hydrogels induces the quick drying when exposed to sunshine. The loss of embedded water will cause the malfunction of photocatalytic water splitting.^[^ ^32^ ^]^ Although the hydrogels can spontaneously absorb the moisture or dew from their external atmosphere, the transport of water from the surface to the interior of the hydrogels is relatively slow, which significantly hinders the water collection. Due to these limitations, a new structure of hybrid hydrogels is highly desired to realize the long‐lasting hydrogen evolution together with an efficient water collection.

Because of its good chemical stability, high mechanical strength, and well thermal stability, polyvinylidene fluoride (PVDF) has attracted a huge attentions in the last decades.^[^ ^33^, ^34^, ^35^ ^]^ Concerning the optical properties, PVDF membranes are well known for their good electrical activity, high diffraction efficiency, and significant nonlinear optical effects. Concerning the thermal properties, PVDF has a low thermal conductivity. Thus, it can effectively scatter the incident light to lower the temperature of hydrogels. Based on the unique optical and thermal properties, PVDF membranes are broadly used for oil–water separation,^[^ ^36^ ^]^ nanogenerators,^[^ ^37^ ^]^ piezoelectric sensors,^[^ ^38^ ^]^ energy harvesting,^[^ ^39^ ^]^ and cooling/heating applications.^[^ ^40^ ^]^ Concerning the optical and thermal analysis of PVDF membranes, the 2D finite‐difference time‐domain simulation, pyranometer, temperature, and infrared (IR) thermal imager are frequently applied. Unlike the hydrophilic hydrogels, PVDF is a well‐known hydrophobic polymer. Based on its hydrophobicity, the introduction of PVDF onto hybrid hydrogels can effectively slow down the water evaporation and maintain the water retention. However, simply coating a dense PVDF layer on the surface will prevent the hydrogen transport from the hydrogels, which will lower the efficiency of the hydrogen evolution. Unlike a dense coating, electrospinning will produce fibers with a nanometer scale and form a porous membrane.^[^ ^40^ ^]^ Thus, the electrospun PVDF membrane prominently slows down the evaporation of water in the hydrogels, and significantly improves its water retention capacity, which prominently extends the life of hydrogen production in the hybrid hydrogels.^[^ ^41^, ^42^, ^43^, ^44^, ^45^ ^]^ In addition, a Janus structure is established by the upper hydrophobic PVDF membrane and the lower hydrophilic hybrid hydrogel. According to previous investigations,^[^ ^46^, ^47^, ^48^, ^49^, ^50^, ^51^ ^]^ the water transport rate from the hydrophobic to the hydrophilic part can be profoundly accelerated. Thus, an efficient collection of water from the surrounding environment, such as dew in the morning, can be realized in the hybrid hydrogels covered with a PVDF membrane if they are used in an outdoor application. It favors for the water storage and prolongs the lifetime of the hydrogen production in the hybrid hydrogels. Therefore, the present hybrid hydrogels covered with electrospun PVDF membrane can simultaneously realize the long‐lasting hydrogen evolution and efficient water collection, suitable for the nonaqueous environment rich in solar resource.

## 2 Results and Discussion

### 2.1 Morphology of the Hybrid Hydrogels Covered with Electrospun PVDF Membrane

**Figure 1:**
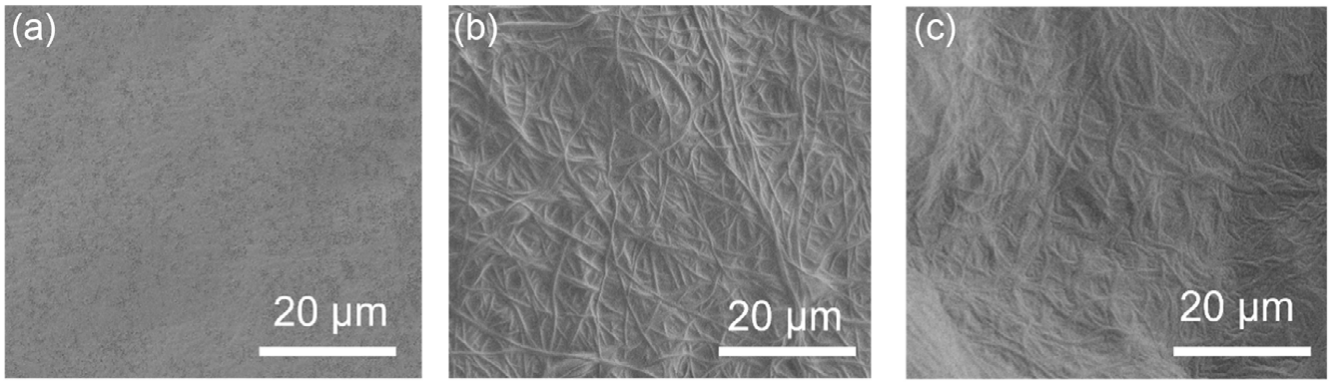
Surface morphology of a) hybrid hydrogels and hybrid hydrogels covered with electrospun PVDF membranes with different thicknesses: b) 48 μm and c) 65 μm.

The surface morphology of the hybrid hydrogels as well as the hybrid hydrogels covered with electrospun PVDF membrane is shown in **Figure** **1**. Compared to the smooth surface of the hybrid hydrogels (Figure 1a), the electrospun PVDF nanofibers can be observed on surface after electrospinning for 2 min (Figure 1b). The membrane thickness is only 48 μm. When the electrospinning time is prolonged to 5 min, the membrane thickness is also increased to 65 μm (Figure 1c). It indicates the successful electrospinning of PVDF on the surface. However, due to the very closed thickness values, no prominent differences between the membranes is observed in the scanning electron microscopy (SEM) images. In addition to SEM images, optical microscopy is also applied to characterize the surface morphology. As shown in Figure S1a, Supporting Information, the surface of the hybrid hydrogels is very smooth. Only a limited number of clusters formed by the g‐C_3_N_4_ nanosheets can be observed. After electrospinning PVDF membranes on the surface, it is obvious that the surface turns rougher and an aggregation of nanofibers is observed on the surface (Figure S1b,c, Supporting Information).

**Figure 2:**
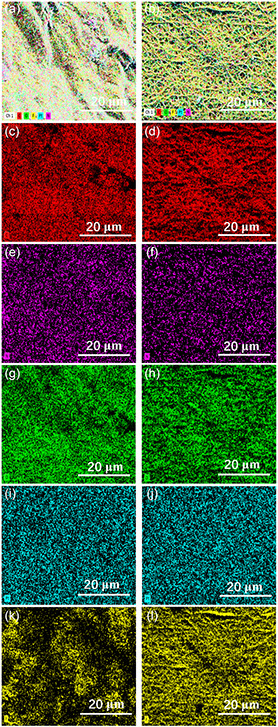
EDS mapping profiles of hybrid hydrogels covered with electrospun PVDF membranes with different thicknesses: a,c,e,g,i,k) 48 μm; b,d,f,h,j) 65 μm.

To further address the successful electrospinning of the PVDF membrane on hydrogels, energy dispersive spectroscopy (EDS) mapping profiles are recorded to investigate the elements present on the surface. Compared to the hybrid hydrogels (Figure S2, Supporting Information), the additional element F can be observed in the hybrid hydrogels covered with electrospun PVDF membranes (**Figure** **2**k,l). Since only PVDF contains the element F, it can be concluded that the PVDF is electrospun on hydrogel surface. In addition, when the PVDF membrane is thickened from 48 to 65 μm, the intensity of element F is more pronounced in the EDS mapping profile, indicating that there is more element F on surface. The amounts of elements in the hybrid hydrogels covered with electrospun PVDF membranes can be found in Table S1, Supporting Information.

### 2.2 Chemical Structure of the Hybrid Hydrogels Covered with Electrospun PVDF Membrane

**Figure 3:**
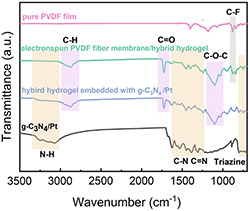
ATR–FTIR spectra of g‐C_3_N_4_/Pt nanosheets (black curve), hybrid hydrogels (purple curve), pure PVDF membrane (pink curve), and hybrid hydrogels covered with electrospun PVDF membrane (green curve).

The chemical structures of the g‐C_3_N_4_/Pt nanosheets, hybrid hydrogels, pure PVDF membrane, as well as hybrid hydrogels covered with electrospun PVDF membrane are probed by attenuating total reflection Fourier transform IR (ATR–FTIR) spectroscopy. In the spectra of g‐C_3_N_4_/Pt nanosheets (black curve in **Figure** **3**), the characteristic peak at 810 cm^−1^ corresponds to the tris‐s‐triazine unit. In addition to that, several consecutive absorption peaks related to the C=N double bonds and C—N single bonds are visible in the range of 1200–1600 cm^−1^. A broad characteristic absorption peak at 3400–3700 cm^−1^ is observed as well, which is attributed to the stretching vibration of N–H groups. In the spectra of hybrid hydrogels (purple curve in Figure 3), the absorption peak related to the C—H stretching vibration is located at 2882 cm^−1^. The stretching vibration peaks of C=O and C—O—C are observed at 1727 and 1112 cm^−1^, respectively. In the spectra of pure PVDF membrane (pink curve in Figure 3), the characteristic peaks at 850 cm^−1^ correspond to the C—F single bonds.

Due to the additional PVDF membrane on the surface, the characteristic peaks related to the C=N bonds and C—N bonds between 1200 and 1600 cm^−1^ in the spectra of the hybrid hydrogels covered with electrospun PVDF membrane (green curve in Figure 3) are not as prominent as those in the spectra of the g‐C_3_N_4_/Pt nanosheets. However, the characteristic absorption peaks of N—H and the tris‐s‐triazine unit from g‐C_3_N_4_/Pt as well as the characteristic absorption peaks of C—F bonds from PVDF are still visible. Therefore, it can be concluded that both g‐C_3_N_4_/Pt and PVDF membranes are introduced into the hydrogels.

In addition to the ATR–FTIR spectra, UV–vis spectra are also applied for the optical analysis. As shown in Figure S3, Supporting Information, due to the presence of g‐C_3_N_4_/Pt nanosheets, the hybrid hydrogels without electrospun PVDF membrane (blue curve) show a prominent absorption in the range of 200–350 nm. When the PVDF membrane is electrospun on the surface, an increase of the absorption is observed not only in the UV range, but also in the visible‐light range (green curve), even in case that the membrane thickness is only 48 μm. This behavior can be attributed to the light absorption by the additional PVDF membrane. Further increasing the thickness to 65 μm (black curve), the absorption is again slightly increased.

### 2.3 Hydrophilicity and Water Transport Capability of the Hybrid Hydrogels Covered with Electrospun PVDF Membrane

**Figure 4:**
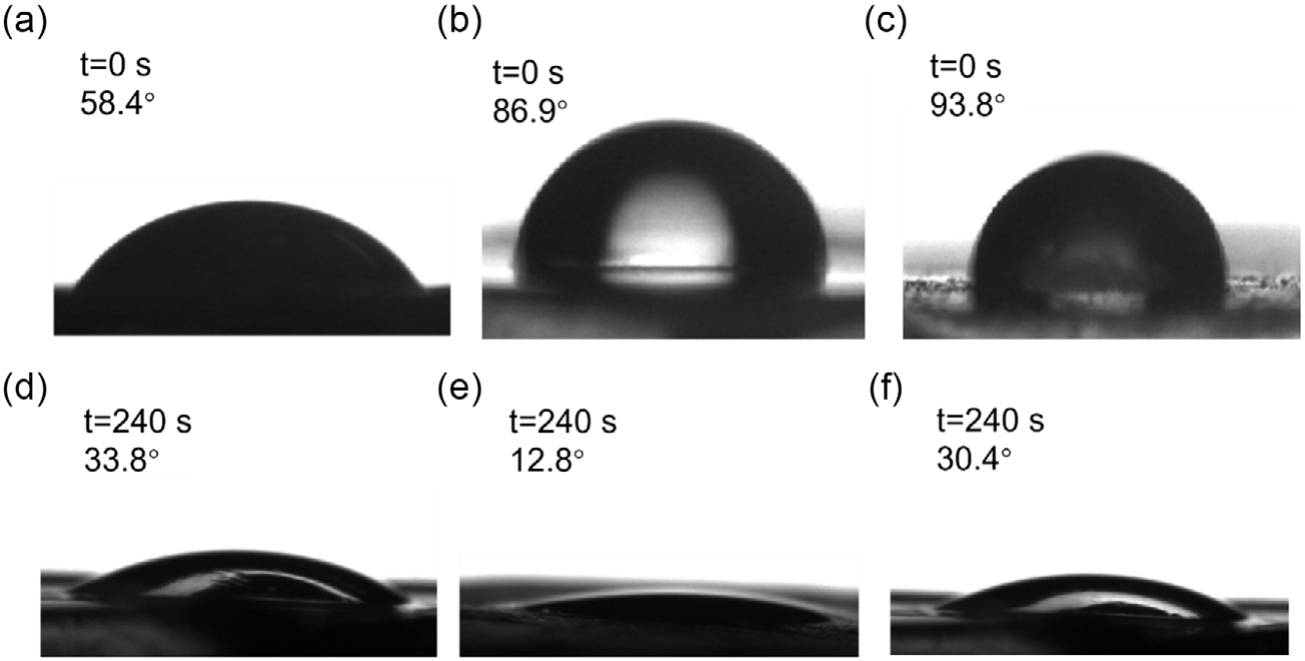
Contact angles of a,d) hybrid hydrogels; b,e) hybrid hydrogels covered with electrospun PVDF membrane (48 μm); and c,f) hybrid hydrogels covered with electrospun PVDF membrane (65 μm) after immediately dropping a water droplet on the surface (*t* = 0 s, top) and 240 s after dropping the droplet (bottom).

The hydrophilicity and water transport capability of the hybrid hydrogels as well as the hybrid hydrogels covered with electrospun PVDF membranes with different thicknesses are investigated by contact angle measurements. As shown in **Figure** **4**a, the contact angle of the hybrid hydrogels is 58.4°, indicating that the hydrogel surface is rather hydrophilic. After electrospinning of the PVDF membrane with a thickness of 48 μm, the hydrophilicity of the surface is reduced due to the introduction of hydrophobic PVDF. The contact angle prominently increases to 86.9°. Further thickening the PVDF membrane to 65 μm increases the contact angle to 93.8°. Because this value is large than 90°, the surface turns from hydrophilic to hydrophobic. Interestingly, although the surface switches to a hydrophobic state after electrospinning PVDF membrane, the wetting time presents a different tendency. After dropping the water droplet on the surface for 240 s, the contact angle of the hybrid hydrogels is still 33.8°. It illustrates that although the surface of the hybrid hydrogels is hydrophilic, the absorption of a water droplet by the hydrogel is relatively slow. However, the contact angle of the hybrid hydrogels covered with electrospun PVDF membrane (48 μm) is only 12.8° after the same time (240 s). Accordingly, more water is absorbed by the hybrid hydrogels when an additional hydrophobic PVDF membrane is on surface. The reason can be attributed to the Janus structure constructed by the hydrophobic PVDF membrane and hydrophilic hybrid hydrogels. It induces the fast transport of water from the hydrophobic to the hydrophilic side.^[^ ^47^, ^48^, ^49^ ^]^ Thus, the contact angle is much smaller than that of the hybrid hydrogels without electrospun PVDF membrane. Further thickening the membrane thickness to 65 μm increases the contact angle again to 30.4° after 240 s. This behavior might be related to the difficult transfer of water molecules when the hydrophobic PVDF membrane is too thick. However, it should be noted that although the thick hydrophobic PVDF membrane slows down the transport of the water droplet, the contact angle is still smaller than that of hybrid hydrogels without PVDF membrane. Thus, the Janus structure indeed plays an important role in the water transport, which can be used for the efficient dew collection in our present investigation.

**Figure 5:**
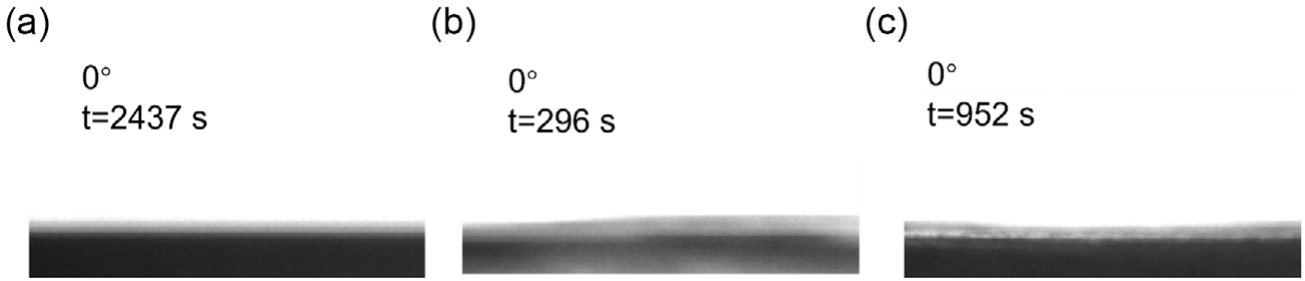
Wetting time of a) hybrid hydrogels and hybrid hydrogels covered with electrospun PVDF membranes with different thicknesses: b) 48 μm and c) 65 μm.

In addition to the contact angle, the wetting time is also measured to investigate the water transport capability. As shown in **Figure** **5**a, the hybrid hydrogels require 2437 s to fully absorb the water droplet. In contrast, the hybrid hydrogels covered with electrospun PVDF membrane (48 μm) require only 296 s, which is 8 times faster than that of hybrid hydrogels. When the electrospun PVDF membrane has a thickness of 65 μm, the wetting time is again profoundly prolonged to 952 s. Although this value is longer than that with thinner PVDF membrane, it is still much shorter than that without PVDF membrane. Such behavior again confirms the important role of the Janus structure for the water transport.

A Janus structure is preferred in our present investigation to realize the fast transport of water from the electrospun PVDF membrane to the hydrogels. Therefore, an efficient dew harvest performance can be realized by the superhydrophobicity of PVDF. To confirm our claim, the contact angles of the electrospun PVDF and PMMA membranes are measured. As shown in Figure S4, Supporting Information, the contact angle of the PVDF membrane is 118.6°, which is much larger than that of the PMMA membrane (74.3°). Therefore, it is beneficial for the construction of a Janus structure in our present investigation. To address the Janus structure constructed by the hydrophobic PVDF membrane and hydrophilic hydrogels, the wetting time and contact angle from the hydrogel side are also measured. As shown in Figure S5a, Supporting Information, the contact angle is 56.6°, indicating that the hydrogel is hydrophilic. However, the wetting time is 2040 s (Figure S5b, Supporting Information). Thus, it can be concluded that the complete absorption of water from the hydrophilic side is very slow. On the contrary, although the contact angles of the PVDF membrane on the hydrogel are as large as 86.9° (48 μm) and 93.8° (65 μm), the wetting times are only 296 s (48 μm) and 952 s (65 μm). Both values are much smaller than that of the hydrogel side. Therefore, it can be confirmed that the present hybrid hydrogels covered with the electropsun PVDF membrane possess a Janus structure.

**Figure 6:**
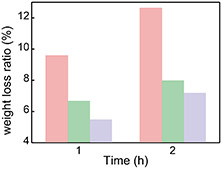
Weight loss of hybrid hydrogels under infrared light illumination: hybrid hydrogels without electrospun PVDF membrane (pink column), hybrid hydrogels covered with electrospun PVDF membranes with thicknesses of 48 μm (green column) and 65 μm (purple column).

In addition, the temporal evolution of the hydrogel weight under IR light illumination is studied to address the water retention capability of the electrospun PVDF membrane. The schematic illustration of the water retention measurements is shown in Figure S6, Supporting Information. The hybrid hydrogels covered with electrospun PVDF membrane (65 μm) present the best water retention capability. After illumination for 1 h, the weight loss is only 5.5% (purple column in **Figure** **6**), meaning that most water remains inside the hydrogels. This observation is attributed to the lower water evaporation rate of the hybrid hydrogels under illumination caused by the hydrophobicity of the PVDF. When the electrospun PVDF membrane has a lower thickness of 48 μm, the water evaporation accelerates in the same scenario. It causes the weight loss to increase to 6.7% (green column in Figure 6). If there is no electrospun PVDF membrane covering the hybrid hydrogel surface, the weight loss is as high as 9.6%, which is almost doubled to that of the hybrid hydrogels covered with a thick electrospun PVDF membrane (65 μm). Such findings illustrate that the hydrophobic PVDF membrane is crucial for a reduction of the water evaporation and a maintenance of the water retention in the hydrogels. When the illumination time is further prolonged to 2 h, the weight loss in the hybrid hydrogels without electrospun PVDF membrane increases to 12.6%, which is also doubled to that of the hybrid hydrogels covered with electrospun PVDF membrane (65 μm).

### 2.4 Photocatalytic Hydrogen Evolution of the Hybrid Hydrogels Covered with Electrospun PVDF Membrane

**Figure 7:**
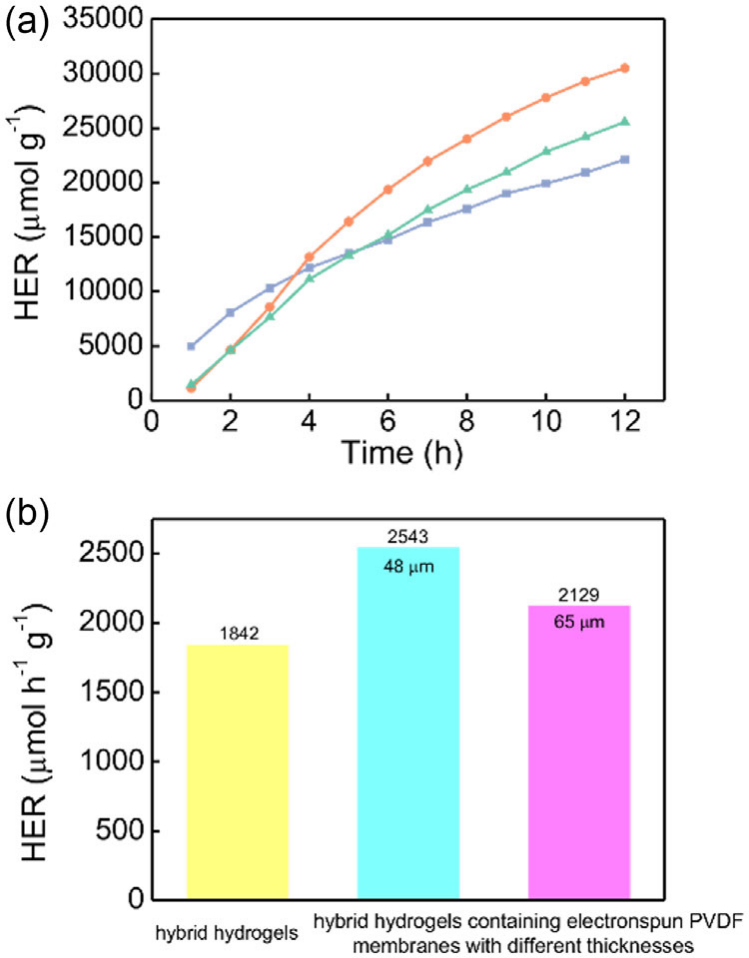
a) Hydrogen evolution rate of hybrid hydrogels (blue curve); hybrid hydrogels covered with electrospun PVDF membrane (48 μm: orange curve and 65 μm: green curve). b) Average HER values in hybrid hydrogels (yellow column); hybrid hydrogels covered with electrospun PVDF membrane (48 μm: cyan column and 65 μm: purple column).

The hydrogen evolution rate (HER) of the hybrid hydrogels as well as of the hybrid hydrogels covered with electrospun PVDF membranes are presented in **Figure** **7**. Unlike the hybrid hydrogels covered with electrospun PVDF membranes, the hybrid hydrogels without PVDF membrane present higher HER values in the first 3 h (blue curve in Figure 7a). The possible reason is the slower transport of hydrogen due to the additional PVDF membrane on the hydrogel surface. Interestingly, when the illumination time is prolonged, the HER values of the hybrid hydrogels covered with electrospun PVDF membranes (orange and green curves in Figure 7a) show a more prominent increase than that of hybrid hydrogels without PVDF membrane. This reversed effect can be attributed to the fast drying process in the hybrid hydrogels without PVDF membrane, which induces the loss of water molecules from the hydrogels, which significantly lowers the efficiency of the photocatalytic water‐splitting process. After 12 h, the HER value of hybrid hydrogels covered with PVDF membrane can reach 30 212 μmol g^−1^ (48 μm) and 25 551 μmol g^−1^ (65 μm), which are 38% and 16% higher than that of the hybrid hydrogels without PVDF membrane. Therefore, it can be concluded that the upper hydrophobic PVDF membrane can well prevent the evaporation of water molecules from the hybrid hydrogels. Due to the good water retention capability, the embedded water molecules can be continuously used for the photocatalytic water splitting by g‐C_3_N_4_/Pt nanosheets. Thus, not only the hydrogen evolution time is significantly prolonged, but also the total amount of produced hydrogen is profoundly increased. It should be noted that although the thicker electrospun PVDF membrane (65 μm) presents the better water retention capability, it also slows down the hydrogen transport from the hydrogels and shields the light illumination exposed on hydrogels. Both effects induce the smaller HER values in case of the thicker PVDF membrane. As shown in Figure 7b, the HER rates in the hybrid hydrogels covered with electrospun PVDF membrane are 2543 μmol h^−1^ g^−1^ (48 μm) and 2129 μmol h^−1^ g^−1^ (65 μm). Both values are much larger than that of the hybrid hydrogels without PVDF membrane (1842 μmol h^−1^ g^−1^).

To address the influence of the membrane thickness on the hydrogen evolution, very thin (20 μm) and thick (150 μm) electrospun PVDF membranes are also prepared and the HERs are measured. Compared to the hybrid hydrogels without covering (black curve in Figure S7, Supporting Information), the hybrid hydrogels covered with very thin electrospun PVDF membrane (20 μm, green curve in Figure S7, Supporting Information) show a visible reduction in the hydrogen evolution. It can originate from two effects. The first one is the covering effect. Although the membrane is very thin, it still shields the visible light and hinders the transport of hydrogen from the interior to the external atmosphere. The second one is the dehydration effect. The thin membrane cannot efficiently slow down the evaporation. Thus, even prolonging the time, the HER values are still low. When the PVDF membrane is thickened to 150 μm (purple curve in Figure S7, Supporting Information), the HER values continue decreasing. Such behavior is caused by the even enhanced shielding effect to visible light and difficulty of the hydrogen transport, although the dehydration is less prominent.

## 3 Conclusion

Long‐lasting hydrogen evolution and efficient dew harvest are realized in the hybrid hydrogels covered with an electrospun PVDF membrane. It reduces the water evaporation rate, which improves the water retention capability of the hydrogels and extends the lifetime for the photocatalytic hydrogen evolution. It can continue working with a high efficiency for 12 h. The HER value of the hybrid hydrogels covered with electrospun PVDF membrane (48 μm) reaches 30 212 μmol g^−1^ in these 12 h, which is 38% higher than that of the hybrid hydrogels without PVDF membrane. Moreover, the hydrophobic PVDF membrane and hydrophilic hybrid hydrogels construct a Janus structure, which favors the directional water transport from the hydrophobic to the hydrophilic part. The wetting time is only 296 s for the hybrid hydrogels covered with the electrospun PVDF membrane (48 μm), which is 800% faster than that of hybrid hydrogels without PVDF membrane. Therefore, it is beneficial for the efficient collection of dew formed on the hydrogel surface in the morning in an outdoor application. Based on the unique properties of long‐lasting hydrogen evolution and efficient dew harvest, the present hybrid hydrogels covered with PVDF membrane can be a promising candidate for hydrogen production in a desert or prairie environment, where is rich in solar resource and lacks water supply.

## 4 Experimental Section

4.1

4.1.1

##### Materials

The monomers di(ethylene glycol) methyl ether methacrylate (MEO_2_MA, purity of 95%), poly(ethylene glycol) methyl ether methacrylate (OEGMA_300_, purity of 95%), and dicyandiamide (C_2_H_4_N_4_, AR) were bought from Aladdin. Ammonium persulfate (APS, purity of 99.99%), N,N,N′,N′‐tetramethyl ethylenediamine (AR) and the cross‐linker N,N′‐methylene bis‐acrylamide (MBA, purity of 99%) were purchased from Shanghai Macklin Biochemical Technology Co., Ltd. The PVDF (average Mw of 180 000 g mol^−1^) and N,N‐dimethylformamide (DMF, gas chromatograph [GC]) were obtained from Macklin. Acetone was purchased from Huzhou Shuanglin Chemical Technology Co., Ltd.

##### Preparation of g‐C_3_N_4_ Nanosheets Loaded with Pt Atoms

The g‐C_3_N_4_ nanosheets were synthesized by the conventional thermal etching method.^[^ ^41^ ^]^ The details for the preparation of the g‐C_3_N_4_ nanosheets can be found in Supporting Information.

##### Preparation of Hybrid Hydrogels Embedded with g‐C_3_N_4_/Pt

The hybrid hydrogels embedded with g‐C_3_N_4_/Pt were prepared by free radical polymerization in deionized water. MBA and APS were used as the cross‐linker and initiator.^[^ ^41^ ^]^ The details for the preparation of the hybrid hydrogels can be found in Supporting Information.

##### Preparation of the Hybrid Hydrogels Covered with Electrospun PVDF Membrane

The electrospinning solution was prepared by dissolving PVDF in the mixed solvents containing DMF and acetone with a mass ratio of 1:1. The mass concentration of the obtained PVDF solution was 10%. After stirring at 45 °C for 4 h, the solution was placed still at room temperature until no bubbles were observed in the solution. The electrospinning system was mainly composed of a high‐voltage power supply, a micro‐syringe pump, a blunt needle, and a plate collector attached to the hybrid hydrogels embedded with g‐C_3_N_4_/Pt nanosheets. The electrospinning solution pumped through the syringe with a speed of 0.3 mL h^−1^. The distance between the needle and the collector was 10 cm, while the voltage was set as 10 kV. By simply adjusting the electrospinning time, the electrospun PVDF membrane with different thicknesses could be obtained on the hydrogel surface.

##### Microscopy Measurements

Field‐emission SEM (ULTRA55, Carl Zeiss SMT Pte. Ltd., Germany) was used to probe the surface morphology of the hybrid hydrogels as well as the hybrid hydrogels covered with electrospun PVDF membrane. The corresponding operating voltages and distance were 8 kV and 10 mm, respectively. Before the measurements, these hydrogels were first dehydrated by a freeze dryer (Lab‐1 A‐50, BIOCOOL, China) and then sputtered with platinum for 120 s by an automated fine coater (JFC‐1600, JEOL, Japan).

##### Characterization of Chemical Structure

The functional groups in the hybrid hydrogels as well as the hybrid hydrogels covered with electrospun PVDF membrane were investigated by ATR–FTIR (Vertex 70 spectrometer, Bruker, USA). The samples were freeze‐dried before measurements. The range of wave numbers was from 600 to 4000 cm^−1^. The number of scans and resolution were 64 and 4 cm^−1^, respectively.

##### Contact Angle Measurements

The contact angle and wetting time in the hybrid hydrogels as well as the hybrid hydrogels covered with electrospun PVDF membrane were characterized by the contact angle tester (DSA 20, Kruss, Germany). The volume of single droplet was set as 3 μL. Each sample was measured three times and the obtained values were averaged to reduce the measurement errors.

##### Water Retention Capability Measurements

The water retention capability was investigated by temporal weight loss of hybrid hydrogels covered with electrospun PVDF membrane under IR light illumination. An IR lamp (Philips, Korea) was used as the light source. The light intensity of the IR lamp was measured by a laser power meter (LP‐3B, Beijing Wuke Photoelectric Technology Co., Ltd.). When the distance between the IR lamp and the sample was 10 cm, the light intensity was 3079.6 W m^−2^. Before the measurements, the weight of the as‐prepared hybrid hydrogels covered with electrospun PVDF membrane was first measured. After illumination for 1 and 2 h, the weight was measured again. To address the influence of the electrospun PVDF membrane to the water retention capability, the hybrid hydrogels without the electrospun PVDF membrane were also measured with the identical protocol.

##### Photocatalytic Hydrogen Evolution Measurements

The photocatalytic hydrogen evolution was measured by a thermal conductivity detector gas chromatography (TCD GC, GC1690, Kulun Technology Co., Ltd., China).^[^ ^32^ ^]^ The details can be found in Supporting Information.

## Conflict of Interest

The authors declare no conflict of interest.

## Supporting information

Supplementary Material

## Data Availability

The data that support the findings of this study are available from the corresponding author upon reasonable request.
