# Supplementary material for: Long‐Lasting Hydrogen Evolution and Efficient Dew Harvest Realized via Electrospinning Polyvinylidene Fluoride Membrane on Hybrid Hydrogels
Source: Small Sci. 2024 May 8;4(7):2400046. doi: 10.1002/smsc.202400046 (PMC11935092; doi:10.1002/smsc.202400046)
Supplement: Supplementary file 1 — Supplementary Material [file SMSC-4-2400046-s001.pdf]

Supporting Information

Long-lasting Hydrogen Evolution and Efficient Dew Harvest Realized via Electrospinning PVDF Membrane on Hybrid Hydrogels

Jie Yu,[1] Mengmeng Chen,[1] Neng Hu,[1] Weijia Wang,[2,] Lin Lei,[2] Huiqing Fan,[2] Peter Müller-Buschbaum[3,*] and Qi Zhong[1,3,*]*

Details for preparation of g-C_3N_4 nanosheets loaded with Pt atoms

The g-C_3N_4 nanosheets were synthesized as follows: Dicyandiamide was calcined at 550 °C for 4 h in air with a ramping rate of 5 °C min^{-1} to obtain lumpy g-C_3N_4, which was further ground into powder by an agate mortar. Then, the g-C_3N_4 powder was calcined at 550 °C for 2 h at a heating rate of 2 °C min^{-1}. The obtained g-C_3N_4 nanosheets were yellow powder. The loading of Pt atoms onto the g-C_3N_4 nanosheets was realized via the $NaBH_4$ reduction strategy. In short, the g-C_3N_4 nanosheets (200 mg) was added into the deionized water with stirring.Then, a H_2PtCl_6 solution (1 mL, 2 mg mL^{-1}) and $NaBH_4$ (400 mg) were sequentially added into the suspension and continue stirring for 30 min. The suspension was filtered and sequentially washed with deionized water and ethanol. After drying in an oven thermo-stated at 80 °C for 12 h, the g-C_3N_4 nanosheets loaded with Pt atoms were obtained. [1]

Details for preparation of hybrid hydrogels embedded with g-C_3N_4/Pt

The hybrid hydrogels embedded with g-C_3N_4/Pt were prepared as follows: Pure g-C_3N_4/Pt nanosheets (10 mg) were first dispersed in the deionized water (5 mL) by ultrasonic radiation for 30 min. Afterward, the monomers $OEGMA_{300}$ (0.714 mL, 5 mmol), MEO_2MA (0.46 mL, 5 mmol), cross-linker MBA (10 mg, 0.05 mmol) were subsequently added to the g-C_3N_4/Pt suspended aqueous solution. After stirring for 30 min, the mixed solution was pumped with nitrogen for 15 min to remove the dissolved oxygen in the solution. After that, the initiator APS (10 mg, 0.044 mmol) and catalyst TEMED (10 μL, 0.067 mmol) were added into the aqueous solution under nitrogen protection. The mixed solution was solidified at 30 °C for 6 h. [1]

Photocatalytic hydrogen evolution measurements

A xenon lamp (HDL-II, Bobei Lighting Electrical Factory, China) was used as the light source for the photocatalytic water splitting. A laser power meter (LP-3B, Beijing Wuke Optoelectronics Technology Co., Ltd, China) was used to measure the light intensity of the xenon lamp. When the distance between the lamp and the hydrogels was 10 cm, the light intensity was 668.8 W m^{-2}. No filter was applied in the light source. Since the hybrid hydrogels and the hybrid hydrogels covered with electrospun PVDF membrane were both measured with the same light source, the influence of the light source can be neglected. The sacrificial agent used in the photocatalytic hydrogen evolution was triethanolamine (TEOA). Before the measurements, the hydrogels were immersed in a mixed solution containing TEOA (4 mL) and distilled water (10 mL) for 48 h. [2]

Before the photocatalytic water splitting measurements, N_2 was injected into the sealed glass tube to establish an N_2 atmosphere. After every interval (1 h), 400 μL of gas was withdrawn from the sealed glass tube by a syringe (1 mL). The photocatalytic hydrogen production was measured by a TCD gas chromatograph (GC1690, Kulun Technology Co., Ltd, China).

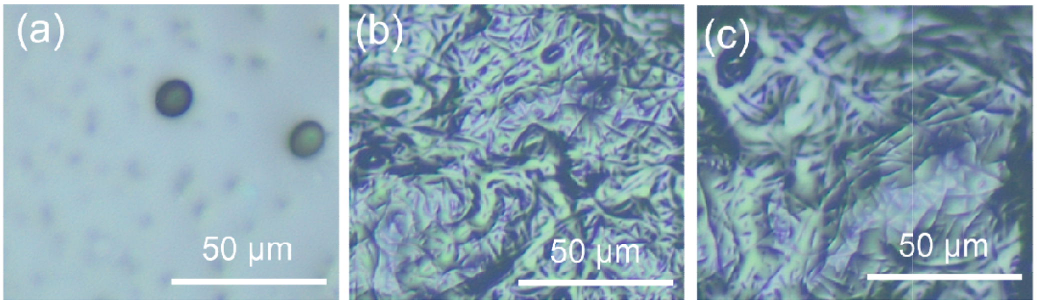

Figure S1. Optical microscopy images of (a) hybrid hydrogels, hybrid hydrogels covered with electrospun PVDF membranes with different thicknesses: (b) 48 μm and (c) 65 μm.

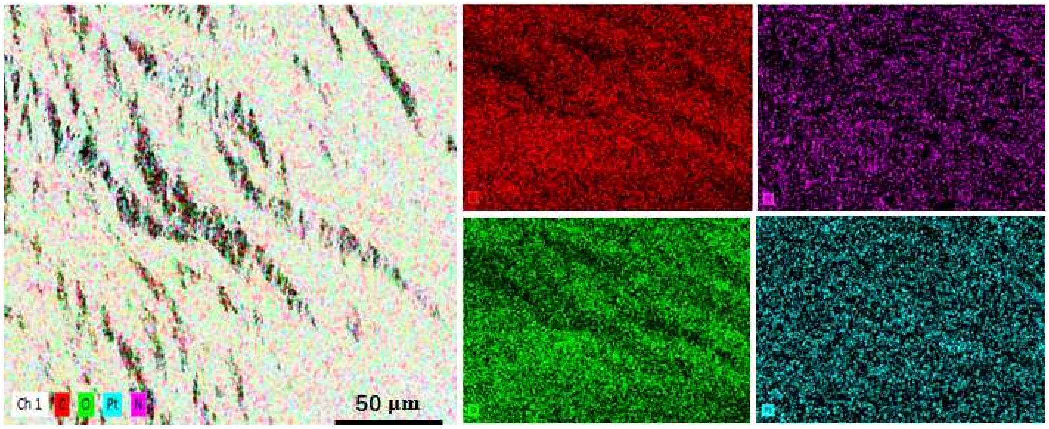

Figure S2.EDS mapping profiles of the hybrid hydrogels.

Table S1. Amounts of elements C, N, O, F and Pt in the hybrid hydrogels covered with electrospun PVDF membranes with different thicknesses.

PVDF membrane thickness	C (%)	N (%)	O (%)	F (%)	Pt (%)
65 μm	43.76	3.81	22.32	29.25	0.86
48 μm	43.73	7.06	43.77	4.83	0.60

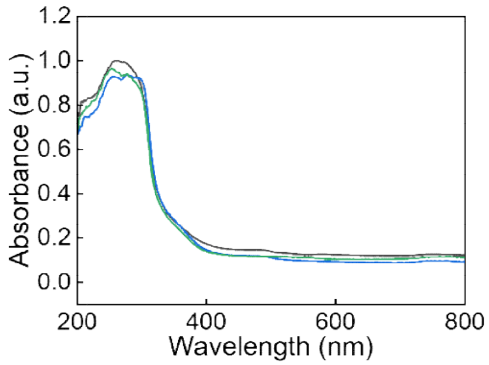

Figure S3. UV-vis spectra of hybrid hydrogels without electrospun PVDF membrane (blue curve), hybrid hydrogels covered with electrospun PVDF membranes with thicknesses of 48 μm (green curve) and 65 μm (black curve).

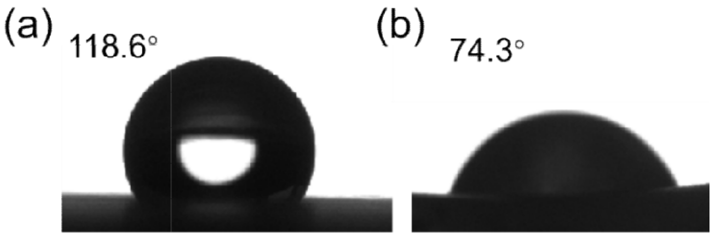

Figure S4. Contact angles of electrospun (a) PVDF and (b) PMMA membranes.

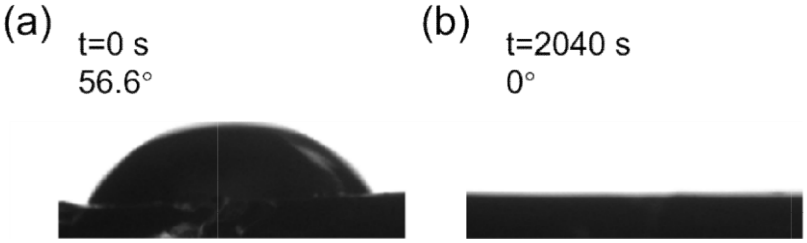

Figure S5. Contact angle andwetting time of the hydrogel side in the Janus structure formed by the hybrid hydrogels and electrospun PVDF membrane.

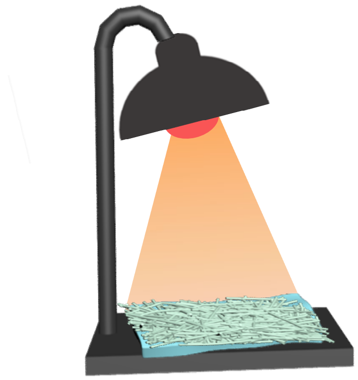

Figure S6. Schematic illustration of the water retention measurements for the hydrogels under infrared light illumination.

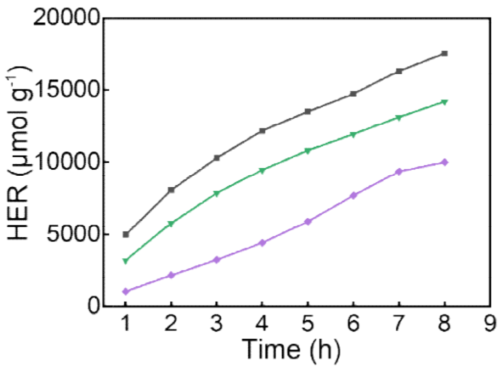

Figure S7. Hydrogen evolution rates (HER) of hybrid hydrogels (black curve), hybrid hydrogels covered with very thin (20 μm, green curve) and very thick (150 μm, purple curve) electrospun PVDF membrane.

Reference

[1] L. Lei, W. J. Wang, C. Wang, H. Q. Fan, A. K. Yadav, N. Hu, Q. Zhong, P. Müller-Buschbaum, *J. Mater. Chem. A.* **2020**, *8*, 23812.

[2] N. Hu, W. J. Wang, L. Lei, H. Q. Fan, Y. Q. Tan, H. Yuan, Z. W. Mao, P. Müller-Buschbaum, Q. Zhong, *Green Chem.* **2021**, *23*, 8969.
